# Smallholder Farmers and Climate Smart Agriculture: Technology and Labor-productivity Constraints amongst Women Smallholders in Malawi

**DOI:** 10.1177/0971852416640639

**Published:** 2017-10-25

**Authors:** Una Murray, Zewdy Gebremedhin, Galina Brychkova, Charles Spillane

**Affiliations:** 1 3D4AgDev Program, Plant & AgriBiosciences Research Centre (PABC), School of Natural Sciences, National University of Ireland Galway, University Road, Galway, Ireland

**Keywords:** Climate change, women smallholders, labor productivity, participatory technology design, agriculture, economic growth

Climate change and variability present a major challenge to agricultural production and rural livelihoods, including livelihoods of women smallholder farmers. There are significant efforts underway to develop, deploy, and scale up Climate-Smart Agricultural (CSA) practices and technologies to facilitate climate change adaptation for farmers. However, there is a need for gender analysis of CSA practices across different farming and cultural systems to facilitate adoption by, and livelihood improvements for, women smallholder farmers. Climate change poses challenges for maintaining and improving agricultural and labor productivity of women smallholder farmers. The labor productivity of many women smallholders is constrained by lack of access to labor-saving technologies and the most basic of farm tools. Poorer smallholders face a poverty trap, due to low agricultural and labor productivity, from which they cannot easily escape without access to key resources such as rural energy and labor-saving technologies. In Malawi, the agricultural system is predominantly rainfed and largely composed of smallholders who remain vulnerable to climate change and variability shocks. Despite the aspirations of women smallholders to engage in CSA, our research highlights that many women smallholders have either limited or no access to basic agricultural tools, transport, and rural energy. This raises the question of whether the future livelihood scenarios for such farmers will consist of barely surviving or “hanging in”; or whether such farmers can “step up” to adapt better to future climate constraints; or whether more of these farmers will “step out” of agriculture. We argue that for women smallholder farmers to become more climate change resilient, more serious attention to gender analysis is needed to address their constraints in accessing basic agricultural technologies, combined with participatory approaches to develop and adapt CSA tools and technologies to their needs in future climates and agro-ecologies.

Gender, Technology
and Development
20(2) 117–148
© 2016 Asian Institute
of Technology
SAGE Publications
sagepub.in/home.nav
DOI: 10.1177/0971852416640639
http://gtd.sagepub.com

**Una Murray**[1]
**Zewdy Gebremedhin**[1]
**Galina Brychkova**[1]
**Charles Spillane**[1]

## Abstract

Climate change and variability present a major challenge to agricultural production and rural livelihoods, including livelihoods of women smallholder farmers. There are significant efforts underway to develop, deploy, and scale up Climate-Smart Agricultural (CSA) practices and technologies to facilitate climate change adaptation for farmers. However, there is a need for gender analysis of CSA practices across different farming and cultural systems to facilitate adoption by, and livelihood improvements for, women smallholder farmers. Climate change poses challenges for maintaining and improving agricultural and labor productivity of women

[1] 3D4AgDev Program, Plant & AgriBiosciences Research Centre (PABC), School of Natural Sciences, National University of Ireland Galway, University Road, Galway, Ireland.

smallholder farmers. The labor productivity of many women smallholders is constrained by lack of access to labor-saving technologies and the most basic of farm tools. Poorer smallholders face a poverty trap, due to low agricultural and labor productivity, from which they cannot easily escape without access to key resources such as rural energy and labor-saving technologies. In Malawi, the agricultural system is predominantly rainfed and largely composed of smallholders who remain vulnerable to climate change and variability shocks. Despite the aspirations of women smallholders to engage in CSA, our research highlights that many women smallholders have either limited or no access to basic agricultural tools, transport, and rural energy. This raises the question of whether the future livelihood scenarios for such farmers will consist of barely surviving or "hanging in"; or whether such farmers can "step up" to adapt better to future climate constraints; or whether more of these farmers will "step out" of agriculture. We argue that for women smallholder farmers to become more climate change resilient, more serious attention to gender analysis is needed to address their constraints in accessing basic agricultural technologies, combined with participatory approaches to develop and adapt CSA tools and technologies to their needs in future climates and agro-ecologies.

Climate change, women smallholders, labor productivity, participatory technology design, agriculture, economic growth

## Introduction

Climate change and increased climate variability are emerging challenges facing agriculture globally (Allen et al., 2014). While climate change impacts on biophysical parameters affecting agriculture and food production are increasingly well understood, the social and gendered impacts of climate change and climate variability remain less well understood (Ashby et al., 2012; Brody, Demetriades, & Esplen, 2008; Jost et al., 2016). There are a multiplicity of efforts underway to climate-proof agricultural production, including through efforts to transition to more "climate-smart agriculture" systems (Lipper et al., 2014). For instance, the The New Partnership for Africa's Development (NEPAD Africa) Africa Climate Smart Agriculture (CSA) Alliance has set a target of 25 million African farmers practicing Climate-Smart Agriculture by 2025.

Climate-smart agriculture is defined by the Consultative Group on International Agricultural Research (CGIAR's) CCAFS program and others as "agriculture that sustainably increases productivity, enhances resilience (adaptation), reduces/removes greenhouse gases (mitigation), and enhances achievement of national food security and development goals" (FAO, 2010; Lipper et al., 2014).

According to FAO (2008), the livelihoods of approximately 2.5 billion people are derived directly from agricultural production systems, either in full- or part-time farming, or in farming households. Sub-Saharan Africa's rural economy is strongly rooted in agriculture as compared to other regions (Livingstone, Schonberger, & Delaney, 2011). Agricultural systems are largely based on smallholder farms which Wiggins and Keats, (2013) defines as being of two hectares or less. Such smallholder farms represent 80 percent of all farms in the sub-Saharan Africa, contributing up to 90 percent of the production in some countries (Wiggins and Keats, 2013). A large percentage of these smallholders are women (Livingstone et al., 2011).[1]

Labor productivity can be defined as the agricultural output per unit labor or the income generated per unit labor. The low labor productivity of smallholder farmers perpetuates rural poverty due to: (a) lack of surplus (income); (b) consistently high time burdens for cultivation and post-harvest tasks; and (c) knock-on impacts on household health and livelihoods. Smallholder women farmers face major labor productivity constraints, particularly where they have limited access to labor-productivity enhancing resources, technologies, or tools (Carrand & Hartl, 2010; Sosovele, 2000).

Labor was identified as a key barrier to achieving equality in productivity across six countries profiled by the World Bank (O'Sullivan, Rao, Banerjee, Gulati, & Vinez, 2014). Labor concerns revolved around women's own labor ability to produce outputs and the quantity and quality of the additional labor women are able to access (i.e., hired or often their own children). Insufficient labor, poor supervision of labor, and family responsibilities are constraints for smallholder farmers. Women's labor is also constrained because of their unpaid work in the care economy, which can vary over their life (e.g., prior to childbirth, childcare, or caring for the elderly) (Peterman, Quisumbing, Behrman, & Nkonya, 2010).

Labor productivity of outdoor workers is expected to be negatively affected by climate change effects such as global warming and increased frequency of extreme (e.g., heat, floods, etc.) weather events (Kjellstrom, Kovats, Lloyd, Holt, & Tol, 2009). Climate change will increase average temperatures but also shift distributions of daily temperature and humidity highs. Such changes will require adaptation by outdoor farm workers to new working cycles, and is likely to have impacts on daily time-use and seasonal labor productivity.

Even in the face of climate change challenges, it is expected that smallholder farmers will continue to play a significant role in agriculture, particularly in developing countries (Wiggins & Keats, 2015). Yet, not all smallholders will remain in agriculture, particularly when faced with adverse climate change impacts. Dorward (2009) describes three main options for smallholders: "hanging in," "stepping up," or "stepping out" of agriculture. "Stepping out" of agriculture into the non-farm economy may be a realistic option for smallholders in areas where climate change is expected to adversely affect agricultural production. Rural youth who do not view farming as a source of sustainable livelihoods are also stepping out of agriculture and rural areas in large numbers (White, 2012). While climate change and climate variability are expected to have an impact on rural-to-urban mobility patterns due to displacement, voluntary migration and/or planned relocation, it should be noted that climate and weather effects are one amongst the many drivers of rural-to-urban migration (Brown, 2008).

The most marginalized smallholders are described as "hanging in," currently barely subsisting from agriculture (Dorward, 2009). Many smallholder households using handheld agricultural tools with limited farm energy and labor constraints fall in this category. Climate change will likely aggravate the risk of food insecurity for such groups. "Stepping up" in agriculture, is where smallholder farmers have better opportunities for agricultural intensification, and to become more engaged in markets. For many smallholders, the lack of options to step up (or step out) of agriculture means that the agriculture sector will remain important for improving food security and rural livelihoods (Diao, Hazell, & Thurlow, 2010; Lipton, 2012; Wiggins & Keats, 2015). In general, CSA practices are being promoted to assist those men and women farmers whose aim is to stay in agriculture.

For farmers who plan to remain in agriculture, gender roles have impacts on both farming and livelihood systems (Quisumbing & Pandolfelli, 2010). Women farmers in particular may not have the same power as men farmers to make important decisions relating to changing agricultural practices. For example, in Malawi, policies and subsidy programs encourage tobacco and maize growing where women are the core source of labor. However, when it comes to income generation, the men sell the produce and make decisions concerning the use of the income earned.

Just as CSA practices may be climate-smart in one context but not in another, similarly they may have different implications for gender roles in different regions and cultural contexts. The resources, knowledge, and capacity required to adopt a new CSA practice can be significant (World Bank, FAO, & IFAD, 2015). In the scale-up and scale-out of CSA practices, gender roles, access to and control of productive assets and power relations need to be factored into design, delivery, and diffusion of each CSA practice so that barriers or opportunities for CSA adoption are better understood. The promotion of CSA practices needs to be underpinned by more rigorous gender and socioeconomic analysis of direct and indirect effects on farmers' livelihoods (Huyer, Twyman, Koningstein, Ashby, & Vermeulen, 2015; Twyman et al., 2015).

In particular, gender analysis can shed light on decision-making relating to specific climate smart agricultural practices, including intra-household bargaining and resource allocation (Doss, 2013; Doss & Meinzen-Dick, 2015). For instance, different smallholders (e.g., men or women-headed households, women in men-headed households) experience different constraints. While such analysis will take time and resources, they are necessary to help avoid the promotion of CSA practices that may be environmentally positive but socioeconomically regressive or maladaptive.

Gender is not the only factor influencing CSA practice adoption. Quisumbing and Pandolfelli (2010) highlight that other socioeconomic parameters, such as age, marital status, education level, and size of landholding can also affect agricultural technology adoption. Understanding why some smallholder farmers are early adopters of CSA practices is important. For instance, it is important to determine whether early adopters are those smallholders who (a) have capital, (b) have a particular ability or power to adopt, or (c) are motivated to change their existing practices.

It is important to ensure that the promotion of climate smart agricultural practices, considered to deliver agro-environmental benefits, does not directly or indirectly generate co-disadvantages that adversely affect the workloads of rural women and children (Giller et al., 2015; Giller, Witter, Corbeels, & Tittonell, 2009). For instance, conservation agriculture may increase the burden of labor on women due to increase in weeding responsibilities, while decreasing the burden of labor on men due to reduction in tillage responsibilities (Kaczan, Arslan, & Lipper, 2013). Different approaches to weed control (e.g., hand hoe weeding versus herbicides) can have differential impacts on the labor and time-use burden on women smallholder farmers (Nyamangara et al., 2014; Thierfelder, Bunderson, & Mupangwa, 2015).

A key challenge relates to how to sustain CSA practices that are based on initial project supports to smallholders, or subsidies that may not reach women farmers. The promotion of planting basins in conservation agriculture programs was found to increase labor demand, yet had no major impact on crop yields, suggestive of a practice that may be unsustainable once project support has finished (Rusinamhodzi, 2015).

For CSA practices to be adopted at scale, women smallholders must have access to relevant inputs and tools (World Bank, FAO, & IFAD, 2015). Yet, women farmers continue to experience barriers to the adoption of new labor-productivity enhancing technologies and practices or CSA practices (Denton, 2002; Sims & Kienzle, 2006a; Sims, Bhatti, Mkomwa, & Kienzle, 2012; UN-Women, 2015). A key question which this article explores is whether women smallholder farmers in Malawi are currently in a position to adopt CSA practices at scale based on current rural energy and technology access levels.

## Methodology

The research data in this article is derived from ongoing fieldwork in the 3D4AgDev project in Malawi, which is a participatory technology development project to develop labor saving tools and technologies with women smallholders. For this article, a systematic review of academic and development literature was conducted on gender, climate change, and labor productivity with a focus on women smallholder farmers. Identification of articles was based on keyword searches. The field research was undertaken through survey questions and focus group discussions between February and March, 2014 in two areas (i.e., Nkhamenya and Kabudula) in Malawi with 1,592 women smallholder farmers to determine their current level of access to labor productivity enhancing tools and technologies, rural energy, and other livelihood assets. The Kabudula Traditional Authority is in Lilongwe district, while the Nkhamenya Traditional Authority falls in Kasungu district. A subset of the 1,592 farmers provided more in-depth information on labor and productivity constraints, including their access to agricultural technologies. Focus group discussions were undertaken with groups of up to 12 women, while research to document a 24-hour recall of time use using activity clocks was also undertaken. Qualitative research followed guidelines for conducting such research (Bryman, 2015; Fraser & Restrepo-Estrada, 1998)

## Results and Discussion

### *Vulnerability of Smallholder Farmers in Malawi to Climate Change and Adverse Weather Events*

In Malawi, agriculture accounts for over 30 percent of the country's gross domestic product (GDP) and employs about 85 percent of the labor force (Mwanakatwe & Kebedew, 2015). Approximately 85 percent of Malawians live in the rural areas and are involved in subsistence farming. Maize is the major staple crop in Malawi, while cash crops such as tobacco (i.e., the major crop for foreign exchange earnings) are also of major importance (World Bank, 2015). Whilst the 2008 census data indicates a total population of 13 million people (National Statistics Office, 2008), as of 2015, the population is estimated at over 16 million (World Bank, 2015). Malawi is classified by the United Nations as a Least Developed Country (LDC), with 54.2 percent of its population classified as poor according to the 2009 SADC Gender Protocol Barometer Baseline Survey (Ngeyi & Pasipau, 2009).

The 2007 United Nations Human Development Report has rated Malawi as one of the most vulnerable countries in sub-Saharan Africa to deleterious impacts of climate change (Malawi Government, 2015). FAO (2012) considers that due to climate change, population growth, urbanization, and environmental degradation, the frequency and intensity of disasters has been largely increasing in Malawi, mostly affecting rural households. The FAO states that prolonged mid-season dry spells, flooding along river basins and poor rainfall distribution are some of the challenges faced by farmers and the agriculture sector in Malawi (FAO, 2012).

The smallholder farming sector and associated rural livelihoods in Malawi are vulnerable to both climate variability and climate change impacts. For instance, our analysis using the EM DAT disaster-monitoring database indicates that Malawi is subject on a recurring basis to livelihood shocks arising from extremes that are climatological (e.g., drought), hydrological (e.g., floods) and/or meteorological (e.g., storms) (Table 1). While such extreme events are responsible for deaths, such events also lead to major disruption of livelihoods and economic losses. For instance, the EM-DAT disaster-monitoring database indicates economic losses of USD 24 million from the 1991 floods in Malawi (Table 1).

Economic analysis suggested that the impacts of droughts can be more severe than those of floods (Pauw, Thurlow, & van Seventer, 2010). As many floods are localized, their economic effects are estimated to be less severe than droughts.

**Table 1.** Livelihood Shocks in Malawi Due to Extreme Natural Events (1990–2015)

| Disaster Subgroup | Disaster Type | Year | No. of Occurrences | Total Deaths | No. of People Affected |
|---|---|---|---|---|---|
| Climatological | Drought | 1990 | 1 | 0 | 2,800,000 |
| Climatological | Drought | 1992 | 1 | 0 | 7,000,000 |
| Climatological | Drought | 2002 | 1 | 500 | 2,829,435 |
| Climatological | Drought | 2005 | 1 | 0 | 5,100,000 |
| Climatological | Drought | 2007 | 1 | 0 | 520,000 |
| Climatological | Drought | 2012 | 1 | 0 | 1,900,000 |
| Hydrological | Flood | 1991 | 1 | 472 | 150,000 |
| Hydrological | Flood | 1995 | 2 | 1 | 1,300 |
| Hydrological | Flood | 1997 | 1 | 0 | 400,000 |
| Hydrological | Flood | 1998 | 1 | 4 | 15,000 |
| Hydrological | Flood | 1999 | 1 | 0 | 2,000 |
| Hydrological | Flood | 2000 | 1 | 0 | 20,000 |
| Hydrological | Flood | 2001 | 2 | 59 | 508,750 |
| Hydrological | Flood | 2002 | 2 | 9 | 396,340 |
| Hydrological | Flood | 2003 | 2 | 12 | 19,500 |
| Hydrological | Flood | 2005 | 1 | 1 | 44,500 |
| Hydrological | Flood | 2006 | 2 | 8 | 16,000 |
| Hydrological | Flood | 2007 | 4 | 2 | 201,965 |
| Hydrological | Flood | 2008 | 1 | 0 | 16,380 |
| Hydrological | Flood | 2010 | 1 | 0 | 21,290 |
| Hydrological | Flood | 2011 | 3 | 4 | 83,587 |
| Hydrological | Flood | 2012 | 2 | 4 | 90,735 |
| Hydrological | Flood | 2013 | 1 | 3 | 33,000 |
| Hydrological | Flood | 2014 | 1 | 0 | 44,850 |
| Hydrological | Flood | 2015 | 1 | 276 | 638,645 |
| Meteorological | Storm | 2005 | 1 | 11 | 8 |
| Meteorological | Storm | 2012 | 1 | 0 | 6,000 |
| Meteorological | Storm | 2015 | 1 | 5 | 350 |

**Source:** Data derived from EM-DAT International Disaster Database (www.emdat.be).

During 2014 and 2015, Malawi experienced floods and droughts that reduced crop production. Indeed, economic growth during 2015 was projected to slow down to 5.5 percent following the late arrival of rains and severe flooding which damaged crops and infrastructure (Mwanakatwe & Kebedew, 2015). The 2015 floods affected 15 out of 28 districts in Malawi, affecting over 1.1 million people. Over 230,000 people were displaced, while 176 were killed and 172 were reported missing. The estimated cost of losses and damages incurred by Malawi floods was USD 335 million, with recovery and reconstruction costs of USD 494 million (Malawi Government, 2015).

The change in timing of rains as well as the frequency of rainfall has negatively affected Malawian labor productivity for on-farm activities. In recent years, farmers are perceiving fewer and later onset of heavy rains with flooding in some key agri-production areas (Coulibaly, Gbetibouo, Kundhlande, Sileshi, & Beedy, 2015). Some areas are experiencing droughts on a persistent basis and this is expected to worsen in the coming years (Kambauwa, Mlamba, Delgado, & Kabambe, 2015). The Malawi Agricultural Sector-Wide Approach 2011–2015 stated that climate change effects, droughts, and floods were the major climatic hazards affecting crop production (Malawi Government, 2011).

## *The Promotion of Climate-Smart Agriculture (CSA) Practices amongst Smallholders in Malawi*

Efforts are underway through government, non-governmental organizations, bilateral donor, and research institutions to enhance the resilience of smallholder farming systems in the face of climate change and climate variability shocks, particularly in the rainfed agriculture sector where productivity is lowest. Such efforts include promotion of greater access to CSA practices (e.g., conservation agriculture, drought tolerant crop varieties and agro-forestry systems). For instance, the Malawian government's Greenbelt Initiative aims to increase the level of irrigation in farming as a key national adaptation measure (Malawi Government, 2015).

The Malawi Agricultural Sector-Wide Approach (2011–2015) promoted conservation farming technologies that build soil fertility, prevent soil erosion, and conserve rain water (e.g., contour ridging, application of manure, preparation of compost, minimum tillage, agro-forestry, box ridges, tractor ploughing to break the hard hoe pan, and use of herbicides as a labor saving technology) (Malawi Government, 2011). The next

Sector-Wide Approach aims to increase agricultural productivity by additionally recognizing gender roles and responsibilities.

The Malawian government's Intended Nationally Determined Contributions (INDC) 2015 Report submitted to the United Nations Framework Convention on Climate Change (UNFCCC's) COP21 provides a roadmap for climate change adaptation and mitigation activities in Malawi (Malawi Government, 2015). Due to the low level of industrial economic activity in Malawi, national greenhouse gas emissions are low (i.e., amounting to 0.04 percent of the total global emissions in 2015). The Malawian INDC emphasizes that "vulnerability and adaptation assessments have shown that most of Malawi's socioeconomic sectors are prone to negative impacts of climate change." Malawi's INDC further identifies gender as a cross-cutting issue to be mainstreamed across all sectors, and highlights that

> [v]ulnerable and disadvantaged groups carry the burden of the impacts of climate change. Women and girls are particularly impacted, as they have to walk further in search of basic commodities for the family such as firewood and water. Yet, women may not have the authority to decide on alternative and climate-resilient solutions for the household. The adaptation interventions proposed in this INDC are meant to enhance gender inclusiveness in the adaptation programs and projects.

Climate change adaptation is a higher priority than mitigation for men and women smallholder farmers in Malawi. Our research in this article focuses specifically on women smallholder farmers in Malawi, and how well they are equipped to adopt CSA practices.

## *Women Smallholders in Nkhamenya and Kabudula Areas of Malawi Have Limited Decision-making Power Over Land*

The agricultural productivity (i.e., yields or financial returns per hectare) of women farmers is often lower than that of men farmers (Croppenstedt, Goldstein, & Rosas, 2013). In an analysis of six countries (Ethiopia, Malawi, Niger, Nigeria, Tanzania, and Uganda), the World Bank (2014) highlighted that women farmers consistently produce less, in monetary terms, per hectare than their men counterparts. The gender-related productivity gap between men and women farmers has been estimated at 25 percent in Malawi (O'Sullivan et al., 2014). A United Nations/World

Bank study in three countries estimated that closing the gender gap in agricultural productivity in Malawi could increase crop production by 7.3 percent, generating a USD 100 million increase in GDP and a USD 90 million increase in agricultural GDP. Closing the gender gap in agricultural productivity in Malawi could potentially lift 238,000 people out of poverty (UN-Women, UNDP, UNEP, World-Bank, 2015).

Women's labor productivity can be negatively affected if they have no control over the land or resources they need to farm (Meinzen-Dick et al., 2011). Analysis of the Malawi Integrated Household Surveys indicates that female ownership of land improves productivity only in women-headed households (Dimova & Gang, 2013). While owning land promotes female decision-making in cash crops, this is only if the woman is also head of the household. According to their analysis, if a household is male-headed and a woman owns the land, the man spouse has less incentive to use the land efficiently. In the Nkhamenya and Kabudula areas of Malawi, our focus group discussions with 187 women smallholder indicate that men typically own land, and that women smallholders have limited decision-making capacity and little control over the land they use.

## *Rural Women and Girls are Engaged in More Tasks Than Men and Boys in Nkhamenya and Kabudula Areas of Malawi*

Labor productivity is closely linked to the division of tasks and duties in households. Gender analysis can help identify who is currently involved in which tasks, and whether this is likely to change under possible climate change, rural livelihood, or agriculture sector scenarios (Chaudhury, Vervoort, Kristjanson, Ericksen, & Ainslie, 2013; Collier & Dercon, 2014). Our field research in Nkhamenya and Kabudula in Malawi highlights the gender role division of labor in the communities of the women smallholders (Table 2). For instance, in rural Malawi caring for children and food crops is typically the woman's task, while production of the tobacco crop is considered a task of both men and women. Selling cash crops is predominantly men's task, even when processing is done by all household members (men, women, boys, and girls). Gender divisions of labor also relate to children (Murray, 2013). For instance, in Malawi our research indicates that girls typically assist in household tasks and food production, while boys assist in construction.

The analysis in Table 2 suggests that women and girls may have greater workloads than boys and men. Focus group discussions indicated

**Table 2.** Gender Division of Labor in Communities in Nkhamenya and Kabudula Areas of Malawi for Rural Livelihood Activities

| Rural Livelihood Activity | Men | Women | Girls | Boys |
|---|---|---|---|---|
| Growing crops for home consumption | | ✓ | ✓ | |
| Growing crops for commercial purposes | ✓ | ✓ | ✓ | ✓ |
| Selling cash crops | ✓ | | | |
| Post-harvest processing of cash crops | ✓ | ✓ | ✓ | ✓ |
| Post-harvest processing of crops for home consumption | | ✓ | ✓ | |
| Care of small livestock (e.g., chicken, pigs) | | ✓ | ✓ | ✓ |
| Care and sale of cattle and goats | ✓ | | | |
| Preparing and managing irrigation land | | ✓ | ✓ | |
| Building houses, sheds, and granaries | ✓ | | | ✓ |
| Collecting water for home use and Construction needs | | ✓ | ✓ | |
| Collecting firewood | | ✓ | ✓ | |
| Cooking | | ✓ | ✓ | |
| Cleaning household and compound | | ✓ | ✓ | |
| Preparing baths for household | | ✓ | ✓ | |
| Taking care of children | | ✓ | ✓ | |
| Washing clothes and kitchen utensils | | ✓ | ✓ | |
| Tool production and/or maintenance | ✓ | | | |
| Income generating activities (e.g., piece work, small businesses) | ✓ | ✓ | | |

**Source:** Data collected from men and women participants in communities in Nkhamenya and Kabudula ($n$ = 374, 50 percent women and 50 percent men) during activity clock and focus group sessions.

that women typically spend 8 to 10 hours a day on agricultural tasks alone, with an additional 5 to 6 hours of housework and other non-agricultural tasks to complete in a given day.

Our research indicates that collecting firewood is predominately a task of the women. Indeed, rural fuelwood for energy (e.g., cooking) is a key area where co-benefits on human health and climate change mitigation can likely be achieved (Hofstad, Köhlin, & Namaalwa, 2009).

The unsustainable use of fuelwood and charcoal, as 97 percent of Malawians rely on biomass energy for cooking fuel, combined with poor agricultural practices is considered to be driving a high rate of deforestation and forest degradation in Malawi (Malawi Government, 2015). Clean energy sources for rural livelihoods are needed in conjunction with sustainable forest management practices to limit deforestation and forest degradation (Malawi Government, 2015).

In some instances, gender divisions are not strict and can change according to circumstances. In the Nkhamenya and Kabudula rural communities, women can assist men in tasks that the rural communities consider as predominantly meant for men, such as collecting water for building sheds and granaries, transporting agricultural produce, and post-harvest processing of tobacco. Our research indicates that in the rural communities of Nkhamenya and Kabudula, men do not participate in predominantly female activities like taking care of children and the household, and planting staple food crops like maize, beans, soya, and groundnuts. Rural women in these areas indicated that men take care of children only when women are sick. This suggests that when gender roles change over time, gender role swapping may not be the norm. Instead gender role changes can be asymmetric, resulting in increased labor burden for women.

Relieving time constraints can lead to significant improvements in women farmers' well-being and livelihoods. For instance, it can improve the nutritional status of the household, specifically for women and children, because women can spend more time on food preparation and feeding and may use less energy in completing their responsibilities. Women can also potentially use the available time for income-generating activities, including small retail businesses or engaging in value addition activities, such as processing and marketing their own produce. The majority of women smallholder farmers involved in our focus group discussions in Malawi confirmed their interest and willingness to start new businesses, if their time could be freed up and they had adequate support.

### *Women Smallholders of All Ages in Malawi Have Extremely Limited Access to Even the Most Basic of Irrigation Technologies*

Irrigation technologies are one climate-smart agricultural practice that are considered important for the climate resilience of smallholder farmers (Burney & Naylor, 2012). Surface and sub-surface irrigation practices can range from low technology approaches involving watering cans and water diversion to higher technology sprinkler and precision irrigation systems. In Malawi, many organizations are promoting smallholder irrigation. Only a small number of studies have examined differentiated adoption dynamics and dissemination approaches between men and women. For instance, a study on the adoption of treadle pumps in Malawi found that women smallholder adopters are more likely to pay for subsidized treadle pumps in cash, whereas men smallholder adopters mostly obtain a loan (Kamwamba-Mtethiwa, Namara, De Fraiture, Mangisoni, & Owusu, 2012). Such findings may indicate that women have less access to financing than men in relation to irrigation technology access.

To determine the extent and nature of irrigation practices, we surveyed 1,589 smallholder women farmers in Nkhamenya and Kabudula areas of Malawi. This revealed that less than half (i.e., 45 percent) of the 1,589 smallholder women farmers used irrigation methods during the dry season (Table 3). Further examination of these responses revealed that the vast majority of farmers claiming to use irrigation methods were using only the most basic of irrigation methods (i.e., watering can), and more advanced irrigation methods (e.g., treadle pumps, drip irrigation, etc.) were rarely being used. Out of 223 respondents who indicated the mode of irrigation that they used, 211 indicated they used a watering can, while 6 used channel pump irrigation, 1 used a treadle pump, and 1 used a solar powered pump.

We also investigated whether younger women smallholders were more likely to employ any form of irrigation methods during the dry season in both areas as compared to older women (Table 4). The results indicate that there is no major difference in the proportion of women

**Table 3.** Irrigation Methods Used by Women Smallholders in Malawi in the Dry Season (Nkhamenya and Kabudula Areas)

| Marital Status | Do Not Use Irrigation Methods (Dry Season) | Use Irrigation Methods (Dry Season) | Number of Respondents |
|---|---|---|---|
| Married | 792<br>54.36% | 665<br>45.64% | 1,457 |
| Single | 81<br>61.36% | 51<br>38.64% | 132 |
| Total | 873 | 716 | 1,589 |

**Source:** Authors' own.
**Note:** Dry season is between April and October.

**Table 4.** Extent of Use of Irrigation Methods in the Dry Season by Different Age Groups of Women Smallholders in Malawi (Nkhamenya and Kabudula Areas)

| Women Smallholder Age Cohort | Do Not Use Irrigation Methods (Dry Season) | Use Irrigation Methods (Dry Season) | Number of Respondents |
|---|---|---|---|
| 16–25 | 120<br>50.63% | 117<br>49.37% | 237 |
| 26–30 | 146<br>53.48% | 127<br>46.52% | 273 |
| 31–35 | 124<br>51.24% | 118<br>48.76% | 242 |
| 36–40 | 134<br>59.56% | 91<br>40.44% | 225 |
| 41–45 | 83<br>50.30% | 82<br>49.70% | 165 |
| 46–55 | 139<br>54.30% | 117<br>45.70% | 256 |
| 56–94 | 128<br>65.98% | 66<br>34.02% | 194 |
| Total | 874 | 718 | 1,592 |

**Source:** Authors' own.
**Note:** Dry season is between April and October.

smallholders using or not using irrigation methods across different age groups. This suggests that women smallholders of all ages are equally constrained from adopting irrigation technologies, and only the most basic of irrigation technologies (i.e., watering can) are employed by women smallholders, irrespective of age.

We also investigated whether the household size of different women smallholders was associated with the extent of irrigation use in the dry season (Table 5). The survey results indicated little or no difference in irrigation methods used across different household sizes.

Overall, our results indicate that the women smallholders in these two districts either had no access to irrigation techniques or had extremely limited access to the most basic of irrigation technologies. This raises significant questions regarding whether women smallholder farmers are currently in any position to adopt irrigation related CSA practices to mitigate the climate change impacts.

**Table 5.** Extent of Use of Irrigation Methods in the Dry Season by Different Household Sizes for Women Smallholders in Malawi (Nkhamenya and Kabudula Areas)

| Household Size of Women Smallholder | Do Not Use Irrigation Methods (Dry Season) | Use Irrigation Methods (Dry Season) | Number of Respondents |
|---|---|---|---|
| Up to 5 persons | 403<br>56.21% | 314<br>43.79% | 717 |
| 6 to 7 persons | 306<br>54.55% | 255<br>45.45% | 561 |
| 8 to 18 persons | 165<br>52.55% | 149<br>47.45% | 314 |
| Total | 874 | 718 | 1,592 |

**Source:** Authors' own.
**Note:** Dry season is between April and October.

## *Despite the FISP, the Majority of Women Smallholders in Nkhamenya and Kabudula Areas of Malawi Do Not Use Inorganic Fertilizers*

A key aim of the Malawi Farm Input Subsidy Programme (FISP) has been to increase the smallholder farmer's access to fertilizers, although the efficiency and impact of the fertilizer subsidy program on income and cropland allocation has been debated (Chibwana, Fisher, & Shively, 2012; Chibwana, Fisher, Jumbe, Masters, & Shively, 2010; Denning et al., 2009; Dorward & Chirwa, 2010; Jayne & Rashid, 2013). Dimova and Gang (2013) have highlighted that in Malawi, the receipt of coupons for the purchase of seeds or fertilizer for maize had a significant efficiency-improving effect on men-headed households and an insignificant effect on women-headed households. If coupons are directed at poorer women-headed households, bartering may take place to alleviate immediate consumption constraints. Fisher and Kandiwa (2014) indicated that the FISP has narrowed the gender gap in relation to modern maize cultivation practices in Malawi.

We investigated the extent of use of fertilizer or manure by women smallholders in the Nkhamenya and Kabudula areas and found that 79.5 percent of women surveyed used fertilizer or manure (Table 6). Our results indicate that while married women were more likely than single women to use fertilizer or manure (Table 6), there were no differences by

**Table 6.** Use of Fertilizer or Manure by Women Smallholders in Malawi (Nkhamenya and Kabudula Areas)

| Marital Status | No, Does Not Use Fertilizer or Manure | Yes, Uses Fertilizer or Manure | Number of Respondents |
|---|---|---|---|
| Married | 191<br>13.31% | 1,240<br>86.41% | 1,435 |
| Single | 40<br>32% | 84<br>67.20% | 125 |
| Total | 231 | 1,324 | 1,560 |

**Source:** Authors' own.

**Table 7.** Extent of Use of Fertilizer or Manure by Different Age Cohorts of Women Smallholders in Malawi (Nkhamenya and Kabudula Areas)

| Women Smallholder Age Cohort | No, Does Not Use Fertilizer or Manure | Yes, Uses Fertilizer or Manure | Number of Respondents |
|---|---|---|---|
| 16–25 | 39<br>16.81% | 191<br>82.33% | 232 |
| 26–30 | 38<br>14.13% | 231<br>85.87% | 269 |
| 31–35 | 43<br>18.53% | 188<br>81.03% | 232 |
| 36–40 | 32<br>14.16% | 194<br>85.84% | 226 |
| 41–45 | 23<br>14.20% | 139<br>85.80% | 162 |
| 46–55 | 31<br>12.65% | 212<br>86.53% | 245 |
| 56–94 | 25<br>13.44% | 161<br>86.56% | 186 |
| Total | 231 | 1316 | 1,552 |

**Source:** Authors' own.

age in relation to the use of fertilizer or manure (Table 7). In addition, our data indicate that use of fertilizer or manure by women smallholders in the survey areas does not substantially differ according to the household size (Table 8).

**Table 8.** Extent of Use of Fertilizer or Manure by Different Household Sizes for Women Smallholders in Malawi (Nkhamenya and Kabudula Areas)

| Household Size of Women Smallholder | No, Does Not Use Fertilizer or Manure | Yes, Use Fertilizer or Manure | Number of Respondents |
|---|---|---|---|
| Up to 5 persons | 63<br>14.69% | 364<br>84.85% | 429 |
| 6 to 7 persons | 122<br>14.81% | 699<br>84.83% | 824 |
| 8 to 18 persons | 46<br>14.98% | 261<br>85.02% | 307 |
| Total | 231 | 1,324 | 1,560 |

**Source:** Authors' own.

While our results indicate that the majority of women smallholders in these two areas used either inorganic fertilizers or farmyard manure, further investigation of the types of manure used among 990 respondents revealed that the majority (i.e., 81.62 percent or 808 of 990 respondents) of women farmers are using farmyard manure. Despite the presence of the FISP in Malawi, our research indicates that only 12.63 percent (125 of 990) of women smallholders in Nkhamenya and Kabudula areas were using inorganic fertilizers. Furthermore, only a fraction of the 990 women smallholders were using green manure/crop residues (i.e., 4.95 percent or 49 of 990 respondents) or compost (0.81 percent or 8 of 990 respondents), which are promoted as climate-smart agricultural practices (Sakala, Kumwenda, & Saka, 2003). This suggests that women smallholders in these areas are badly positioned in relation to adoption of climate-smart agriculture practices promoted through initiatives such as the Africa CSA Alliance.

## *Women Smallholders in the Nkhamenya and Kabudula Areas of Malawi Remain Highly Dependent on the Low-labor Productivity Hand Held Hoe*

The labor constraints facing women smallholder farmers arise from a range of reasons, including poverty; low levels of education and awareness of the importance of improved agricultural tools; use of poorly manufactured tools; cultural perceptions limiting the adoption of tools and implements; lack of linkages with local toolmakers; and inadequate market research by tool producers, particularly in relation to tools used by women smallholders with limited purchasing power (Tripathi et al., 2012).

Agricultural tools will be important for adopting and adapting new CSA practices to respond to climate change. For instance, Bernier et al. (2013) highlights that a key constraint for women's tree planting lies in digging holes, where women may lack access to the necessary agri-tools as well as the labor required to dig the larger holes needed to ensure better tree survival. This makes it difficult for women to fence and protect investments in trees and adapt agro-forestry climate-smart practices.

In line with other research, (Kienzle, Ashburner, & Sims, 2013; Kienzle & Sims, 2014; Sims & Kienzle, 2006b), our research in the Nkhamenya and Kabudula areas indicates that most of the 1,600 women smallholders surveyed have limited access to farm energy, mechanization and the most basic of agricultural and agri-processing tools. The majority of these smallholders are using very basic labor-intensive agricultural hand tools for onerous tasks, such as weeding, planting, harvesting, and crop/food processing. With minimal access to alternative energy sources (i.e., draught animals or mechanized farm equipment), these smallholders remain largely dependent on human labor for transport, cultivation, and agri-processing.

It is argued that access to energy and agricultural tools in rural areas can directly increase agricultural productivity for women smallholder farmers (Adkins, Oppelstrup, & Modi, 2012; Deichmann, Meisner, Murray, & Wheeler, 2011; UNDP, 2004). However, for many smallholders not much has changed in the past decades. A 1997 review of available tools indicated that the hoe was the most commonly used tool in Sub-Saharan, Southern, and West Africa (IFAD, 1997). Our 2015 survey of smallholder farmers in the Nkhamenya and Kabudula areas indicates that the majority of women smallholder farmers only have access to the traditional hoe for all core farming activities in cultivation and harvesting. The core hand tool used by the women farmers surveyed is the hand hoe, used for all land clearing, ridging, planting, and weeding activities. Where the hoe does not work, the women rely on other small tools or their hands and legs for planting and weeding. These alternatives are labor intensive, inefficient, and time-consuming, in addition to causing adverse physical effects.

## *Women Smallholders Face Major Constraints in Access to Draught Animals in the Nkhamenya and Kabudula Areas of Malawi*

The majority of smallholder farming communities in the Sub-Saharan Africa experience significant "energy poverty" (Szabó, Bódis, Huld, & Moner-Girona, 2013). For instance, 85 percent of Malawi's 15 million population lives in rural areas, of which only 1 percent has access to electricity. Both human and animal power continue to constitute significant sources of rural energy in many developing countries (Fuller & Aye, 2012). After biomass (e.g., fuel wood), these remain the most important energy sources for many rural populations. While there is much emphasis on renewable energy, including in the context of global goals for "sustainable energy for all" (Rogelj et al., 2013), it is important to recognize that the percentage of energy contributed by human and animal power is estimated at twice that of wind power and 13 percent of hydro power, the largest renewable energy sources (Fuller & Aye, 2012).

While substitution of human labor (especially where associated with drudgery) with alternative on-farm energy sources and systems (e.g., draught animals, mechanization) is desirable for the rural millions in poverty, the reality is that such substitution pathways are not being built at the pace and scale that is needed (Baudron et al., 2015). Hence, there are compelling arguments for improving the efficiency of hand, foot, and animal-powered equipment and tools for women smallholders. While animal traction is often posited as a possible source of farm energy for smallholders, there are significant gender issues associated with women smallholders gaining access to animal traction. Doss has highlighted the need for greater emphasis on how women and men access mechanization (Doss, 2001). Indeed, even when animal power is used by women smallholders, they tend to own fewer draught animals (which are a relatively large capital investment). In addition, ploughing with draught animals in many cultures is considered a male task, although there are instances when women do use draught animals for ploughing (Doss, 2001).

We investigated the extent of access to draught animals by women smallholders in the Nkhamenya and Kabudula areas and found that 16.3 percent of the women smallholders had access to a draught animal (Table 9), with no consistent or major differences observed across age (Table 10) or household size (Table 11).

Overall, our survey indicates that the majority of women smallholders in the areas surveyed do not have access to a draught animal. Further investigation of the social networks of the women smallholders was

**Table 9.** Access to a Draught Animal among Women Smallholders in Malawi (Nkhamenya and Kabudula Areas)

| Marital Status | No Access to a Draught Animal | Access to a Draught Animal | Number of Respondents |
|---|---|---|---|
| Married | 1,211<br>83.17% | 244<br>16.76% | 1,456 |
| Single | 116<br>88.55% | 15<br>11.45% | 131 |
| Total | 1,327 | 259 | 1,587 |

**Source:** Authors' own.

**Table 10.** Access to a Draught Animal among Different Age Groups of Women Smallholders in Malawi (Nkhamenya and Kabudula Areas)

| Women Smallholder Age Cohort | No Access to a Draught Animal | Access to a Draught Animal | Number of Respondents |
|---|---|---|---|
| 16–25 | 211<br>89.03% | 26<br>10.97% | 237 |
| 26–30 | 222<br>81.62% | 50<br>18.38% | 272 |
| 31–35 | 205<br>84.71% | 37<br>15.29% | 242 |
| 36–40 | 192<br>85.71% | 31<br>13.84% | 224 |
| 41–45 | 132<br>80.00% | 33<br>20.00% | 165 |
| 46–55 | 212<br>82.81% | 44<br>17.19% | 256 |
| 56–94 | 156<br>80.41% | 38<br>19.59% | 194 |
| Total | 1,330 | 259 | 1,590 |

**Source:** Authors' own.

conducted to assess whether they know anyone in their community who owns draught animals and/or farm machinery. Out of the 145 women smallholders who provided answers to this question, 106 (i.e., 73 percent) knew someone who owned a tractor, while 24 (i.e., 16.6 percent) knew someone who owned an ox cart and 14 (i.e., 9.7 percent) knew someone who owned a plough. This suggests that while there

**Table 11.** Access to a Draught Animal by Different Household Sizes for Women Smallholders in Malawi (Nkhamenya and Kabudula Areas)

| Household Size of Women Smallholder | No Access to a Draught Animal | Access to a Draught Animal | Number of Respondents |
|---|---|---|---|
| Up to 5 persons | 608<br>84.92% | 107<br>14.94% | 716 |
| 6 to 7 persons | 466<br>83.07% | 95<br>16.93% | 561 |
| 8 to 18 persons | 256<br>81.79% | 57<br>18.21% | 313 |
| Total | 1,330 | 259 | 1,590 |

**Source:** Authors' own.

may be at least one tractor, ox cart or plough in their community, there are significant barriers for women to access labor-productivity enhancing technologies.

## *Women Smallholders in the Nkhamenya and Kabudula Areas of Malawi Have Limited Access to Mechanized Rural Transport*

Women and girls in rural areas of Sub-Saharan Africa, including rural Malawi, commonly face mobility challenges because of patriarchy. In addition to poor roads and inadequate transport, Porter (2011) has highlighted how attitudes about women's mobility limits their access to markets, education, and health services (Porter, 2011). Despite the Malawi National Transport Policy, our research indicates that women smallholders in Nkhamenya and Kabudula areas have limited access to rural public transport with only two women out of 1,589 women reporting that public transportation is their main transport source for daily activities (Table 12). The vast majority (i.e., 83.8 percent) of women smallholders surveyed consider walking as their main transportation mode for daily activities, with 13 percent considering cycling as their main form of transportation (Table 12). A small percentage (i.e., 3 percent) of married women cited a motorcycle as their main source of transport (Table 12). In general, the proportion of women using different forms of transport do not differ substantially across age (Table 13) and household size (Table 14), indicating that access to mechanized transport is constrained across all ages and household sizes of women smallholders.

**Table 12.** Transportation Sources for Daily Activities of Women Smallholders in Nkhamenya and Kabudula Regions of Malawi

| Marital Status | Bicycle | Motorcycle | Public Transport | Walking | Number of Respondents |
|---|---|---|---|---|---|
| Married | 194<br>13.32% | 49<br>3.36% | 2<br>0.14% | 1,212<br>83.18% | 1,457 |
| Single | 13<br>9.85% | 0<br>0.00% | 0<br>0.00% | 119<br>90.15% | 132 |
| Total | 207 | 49 | 2 | 1,331 | 1,589 |

**Source:** Authors' own.

**Table 13.** Transportation Sources for Daily Activities among Different Age Groups of Women Smallholders in Malawi (Nkhamenya and Kabudula Areas)

| Women Smallholder Age Cohort | Bicycle | Motorcycle | Public Transport | Walking | Number of Respondents |
|---|---|---|---|---|---|
| 16–25 | 31<br>13.08% | 8<br>3.38% | 0<br>0.00% | 198<br>83.54% | 237 |
| 26–30 | 58<br>21.25% | 8<br>2.93% | 0<br>0.00% | 207<br>75.82% | 273 |
| 31–35 | 36<br>14.88% | 12<br>4.96% | 0<br>0.00% | 194<br>80.17% | 242 |
| 36–40 | 27<br>12.00% | 9<br>4.00% | 0<br>0.00% | 189<br>84.00% | 225 |
| 41–45 | 25<br>15.15% | 7<br>4.24% | 1<br>0.61% | 132<br>80.00% | 165 |
| 46–55 | 13<br>5.08% | 7<br>2.73% | 0<br>0.00% | 236<br>92.19% | 256 |
| 56–94 | 17<br>8.76 | 0<br>0.00% | 1<br>0.52% | 176<br>90.72% | 194 |
| Total | 207 | 51 | 2 | 1,332 | 1,592 |

**Source:** Authors' own.

## Conclusions

At present, women smallholder farmers in Nkhamenya and Kabudula have extremely limited access to rural energy and labor-productivity enhancing innovations. Routes out of poverty for smallholder rural

**Table 14.** Transportation Sources for Daily Activities by Different Household Sizes for Women Smallholders in Malawi (Nkhamenya and Kabudula Areas)

| Household Size of Women Smallholder | Bicycle | Motorcycle | Public Transport | Walking | Number of Respondents |
|---|---|---|---|---|---|
| Up to 5 persons | 105<br>14.64% | 21<br>2.93% | 1<br>0.14% | 590<br>82.29% | 717 |
| 6 to 7 persons | 72<br>12.83% | 16<br>2.85% | 0<br>0.00% | 473<br>84.31% | 561 |
| 8 to 18 persons | 30<br>9.55% | 14<br>4.46% | 1<br>0.32% | 269<br>85.67% | 314 |
| Total | 207 | 51 | 2 | 1,332 | 1,592 |

**Source:** Authors' own.

communities will require a swathe of innovations to improve labor productivity in agricultural systems, as well as to generate income (Chand, Prasanna, & Singh, 2011). A key challenge is how to enable smallholders to increase their income and agricultural production, while at the same time reducing the labor burden on women and children.

While ambitions to encourage smallholders (including women smallholders) to adopt climate-smart agricultural practices at scale are laudable, our research indicates that women smallholders in the Nkhamenya and Kabudula areas of Malawi have extremely limited access to agricultural productivity-enhancing inputs and resources. Women farmers not only lack capital but also many resources such as animal power, irrigation, and credit. Even if they are motivated to adopt CSA practices, they may not have the decision-making power to embrace such practices. This poses major challenges for women smallholders to adopt climate-smart practices currently being promoted (e.g., by the Africa CSA Alliance) in Malawi. Our research raises the question of whether (and how) such smallholder farmers can be reached by CSA practices for climate change adaptation, and whether (and how) such CSA practices will be developed and locally adapted to meet the needs of women smallholders.

If CSA practices, particularly novel or unfamiliar ones, are expected to be adopted by women smallholder farmers, there are significant knowledge, technology, energy, and capacity building gaps that must be bridged before adoption can be considered by women farmers. In situations where they have limited access to extension, financing, and other support services for adoption of new agricultural productivity-enhancing options, the likelihood of CSA adoption may remain low. We consider that participatory technology development and adaptation approaches are needed to increase the extent of access and utility of CSA practices to women smallholder farmers (Ashby & Sperling, 1995; Gupta, 2013). An analysis of the current situation of men and women farmers (i.e., their current roles, access to resources and technologies, and their decision-making power) is necessary, so that both women and men farmers can better identify the challenges and opportunities they face in relation to climate change adaptation.

As the effects of climate change intensify, rural and urban "Green Economy" markets is likely to expand for products and services that support climate resilience (Nhamo, 2013). From a climate justice perspective (Jafry, 2012), it is critical that the poor and women are not marginalized in green growth and sustainable energy pathways due to the general failure to integrate development with environmental goals (Abdallah, Bressers, & Clancy, 2015; Resnick, Tarp, & Thurlow, 2012).

## Acknowledgments

The authors from the 3D4AgDev program at the National University of Ireland, Galway are grateful for financial support from the Bill and Melinda Gates Foundation (BMGF) Grand Challenges Phase I Program, from the Deutsche Gesellschaft für Internationale Zusammenarbeit (GIZ) GmbH, and from the CGIAR Research Program (CRP) on Climate Change, Agriculture and Food Security (CCAFS) led by the International Center for Tropical Agriculture (CIAT). Research work in Malawi was conducted in partnership with CIAT and Concern Malawi under a research agreement with the National University of Ireland, Galway. We are extremely grateful for the logistical and project support offered by partners at CIAT Malawi, Concern Worldwide in Malawi, Catholic Relief Services, and the Mzuzu Catholic Development Commission in Malawi (CADECOM) services.

## Note

1. Agriculture remains the largest sector for women's employment in Oceania, Southern Asia, and sub-Saharan Africa, with approximately 60 percent of women employed in this sector (ILO, 2013).
